# Decoding the Structural Complexity of Viral RNAs with SHAPE to Guide Antiviral Therapeutics

**DOI:** 10.3390/v18050543

**Published:** 2026-05-08

**Authors:** Laura Broglia, Camilla Canale, Andrea Vandelli, Gian Gaetano Tartaglia, Riccardo Delli Ponti

**Affiliations:** 1 RNA Systems Biology Lab, Center for Human Technologies, Istituto Italiano di Tecnologia, Via Enrico Melen, 83, 16152 Genova, Italy; 2 Department of Pharmacy, University of Naples Federico II, 80131 Naples, Italy

**Keywords:** RNA viruses, RNA secondary structure, SHAPE chemical probing, SHAPE-MaP, RNA structurome, RNA-targeted therapeutics

## Abstract

RNA viruses encode multiple layers of regulatory information within their genomes, extending beyond their protein-coding sequences. Through local secondary structures and long-range RNA–RNA interactions, viral RNAs control essential steps of the viral life cycle, including translation, replication, genome cyclization, packaging, and evasion of host defenses. Over the last two decades, chemical probing approaches—particularly Selective 2′-Hydroxyl Acylation analyzed by a primer extension (SHAPE) and its high-throughput derivatives—have transformed our ability to investigate these structures at a single nucleotide resolution and on a genome-wide scale. These technologies have revealed that viral genomes are highly structured and contain numerous functional RNA elements within untranslated regions as well as coding sequences. In this review, we summarize the main experimental strategies used to profile viral RNA architecture, with a focus on SHAPE-based methodologies and complementary approaches. We then discuss the major classes of functional RNA structures identified across diverse viral families, focusing on elements involved in translation and replication, such as internal ribosome entry sites (IRES) and cyclization elements, as well as other functional structures, including XRN1-resistant and frameshifting elements. Finally, we examine how structure-guided analyses are opening new avenues for antiviral intervention, including antisense oligonucleotides, small molecules, and RNA-degrading chimeras. Together, these advances highlight the viral RNA structure as both a key determinant of virus biology and a promising target for therapeutic innovation.

## 1. Introduction

Single-stranded RNA (ssRNA) viruses represent a major proportion of known viral genera, encompassing important human and animal pathogens such as hepaciviruses, coronaviruses, and flaviviruses [1,2]. The genome of positive-sense ssRNA (ssRNA(+)) acts directly as messenger RNA upon infection, serving simultaneously as a template for translation and as the genetic material for replication. By contrast, negative-sense ssRNA viruses require an RNA-dependent RNA polymerase to generate complementary positive-sense RNA intermediates, which serve as templates for viral mRNA transcription and genome replication. Because the positive-sense genome serves as the immediate substrate for both translation and replication, it provides a direct model for studying how primary RNA architecture dictates early-stage host–virus interactions without the intermediary step of viral transcription.

**Figure 1:**
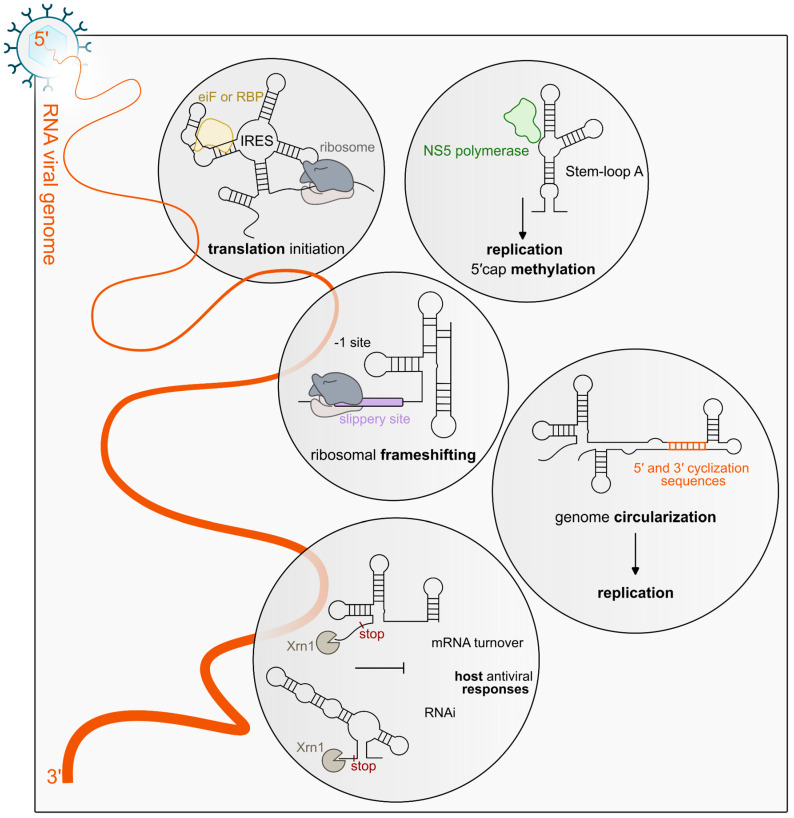
The functional RNA structural elements in viral genomes. Viral RNA genomes contain structured elements that regulate key steps of the viral life cycle. At the 5′ end, internal ribosome entry site (IRES) structures recruit ribosomes and host eukaryotic initiation factors (eIFs) or RNA-binding proteins (RBPs) to promote translation initiation (e.g., picornaviruses and Hepatitis C virus). Structured stem-loop elements can also interact with viral polymerases, such as NS5, to facilitate RNA replication and 5′ cap methylation (e.g., flaviviruses). Within coding regions, programmed ribosomal frameshifting signals—typically composed of a slippery sequence followed by a downstream RNA structure—induce ribosome pausing and allow alternative protein production (e.g., coronaviruses and retroviruses). Long-range interactions between the 5′ and 3′ cyclization sequences (CS) promote genome circularization and replication (e.g., the Dengue and Zika viruses). In addition, highly structured RNA elements can stall the host exonuclease XRN1, generating RNA fragments that modulate RNA turnover and host antiviral responses (e.g., flaviviruses).

Beyond the linear nucleotide sequence, RNA viral genomes encode information through intramolecular base pairing and long-range interactions, which generate distinct secondary and tertiary structures that are essential for efficient replication, translation, and genome packaging [3,4] (Figure 1). Such structured elements act as molecular hubs that recruit host and viral proteins, mediate genome circularization, promote genome compaction, and coordinate the switch between translation and replication [3,5,6,7] (Figure 1). A variety of viral RNA secondary structures have been characterized to date, performing diverse regulatory functions throughout the viral life cycle. One major class of structured RNA elements regulates viral translation. A well-known example is the internal ribosome entry site (IRES), a highly structured RNA element that directly recruits the translation machinery, allowing viral RNAs to initiate translation even when cellular cap-dependent translation is inhibited during infection [8,9] (Figure 1). Although IRES elements are most commonly located in the 5′ untranslated region (UTR), they can also occur downstream of the start codon in some viral genomes [9,10].

Other viral RNA structures contribute to the evasion of host antiviral defenses. In many mosquito-borne flaviviruses (MbFVs), including the Zika virus (ZIKV), Dengue virus (DENV), and West Nile virus (WNV), structured RNA elements stall the cellular 5′–3′ exonuclease XRN1, leading to the accumulation of subgenomic flaviviral RNAs (sfRNAs) during infection [11,12,13,14]. This protection from XRN1-induced degradation is mediated by XRN-1-resistant elements (xrRNAs), typically formed by rigid stem-loop (SL) elements or dumb bell (DB) structures that act as physical barriers to exonuclease progression [9,15,16] (Figure 1). The resulting sfRNAs function as regulatory molecules that interfere with host antiviral responses by dysregulating the RNA decay pathways and sequestering host RNA-binding proteins involved in immune signaling [14,17,18].

RNA structures can also regulate viral gene expression and coding capacity. A prominent example is the programmed ribosomal frameshifting signal, which typically consists of a slippery sequence followed by a downstream RNA structure that induces ribosome pausing and promotes a shift in the reading frame, allowing the production of alternative viral proteins from the same RNA sequence [19,20] (Figure 1).

Beyond local structural motifs, many RNA viruses also rely on long-range intragenomic RNA–RNA interactions to coordinate different stages of their replication cycle. For example, 3′ cap-independent translation enhancers (3′ CITEs)—structured RNA elements composed of stem-loops, pseudoknots, or tRNA-like folds—recruit the eIF4F complex to promote translation despite being located at the 3′ end of the viral genome [21,22,23,24]. These elements function through long-distance RNA–RNA interactions that bring the 3′ CITE into proximity with the 5′ end, enabling efficient ribosome loading [25]. In flaviviruses, complementary interactions between the 5′ and 3′ cyclization sequences (CS) drive genome circularization, acting as a structural switch that represses translation while promoting viral replication [26] (Figure 1).

Together, these examples illustrate how RNA structures encode multiple regulatory layers within viral genomes, controlling translation, replication, immune evasion, and coding potential. Notably, many of these functional elements are frequently located within untranslated regions (UTRs), which serve as major hubs for structural regulation of viral RNA.

Determining the RNA structure is essential for understanding its mechanisms of action and requires experimental approaches capable of probing the RNA architecture at nucleotide resolution. Chemical probing approaches—particularly Selective 2′-Hydroxyl Acylation analyzed by a primer extension (SHAPE)—have become powerful tools to investigate the RNA structure at single nucleotide resolution [3,27,28]. This technique exploits reagents that react with the 2′-hydroxyl group of conformationally flexible nucleotides, forming covalent adducts that are detected during reverse transcription, where they cause termination or mutation events. By quantifying these modifications, SHAPE provides a reactivity profile that reflects local RNA flexibility and can be used to infer secondary structure models. For example, SHAPE analysis of the HIV-1 genome revealed differentially structured regions linked to viral gene expression and replication control [29]. When coupled with next-generation sequencing technologies, SHAPE has enabled genome-wide mapping of viral RNA structures, revealing that many RNA virus genomes are highly structured and that these conformations play critical roles in viral fitness and replication [28,30,31,32,33]. Beyond classical SHAPE, derivative and complementary probing strategies have been developed to capture the RNA structure. Genome-wide secondary structure profiles, combined with structural entropy, can reveal functional structures in viral genomes, several of which reduced viral fitness and virulence when mutated [7,34].

In this review, we summarize recent technological advances in RNA structure probing and highlight how these approaches have expanded our understanding of functional RNA elements in viral genomes. Moreover, we discuss how the identification of novel regulatory structures has provided new insights into viral fitness, replication, and pathogenicity, and we examine the current challenges associated with accurately profiling RNA secondary structures in complex viral systems. Lastly, we explore emerging strategies for targeting viral RNA structures as a novel avenue for antiviral drug development, illustrating how structural knowledge can be translated into therapeutic innovation.

## 2. Experimental Approaches to Profile Viral RNA Structure

RNA molecules can adopt defined secondary (base pairing) and tertiary (three-dimensional folding) structures, underlying their functional versatility [35,36]. However, experimentally resolving RNA base pairing, particularly in long and complex molecules, has been traditionally challenging and often relied on low-throughput approaches such as crystallography. A major conceptual advance came with the development of SHAPE by Merino et al. [37], which enabled the nucleotide-resolution probing of the RNA structure based on local flexibility. This approach has since been widely applied to investigate the structures of diverse single-stranded RNA (ssRNA) viral genomes [38,39,40,41] (Table 1).

Before the development of SHAPE, RNA structure probing primarily relied on chemical footprinting using reagents such as kethoxal, Dimethyl Sulfate (DMS), and 1-cyclohexyl-3-(2-morpholinoethyl)carbodiimide metho-p-toluenesulfonate (CMCT), often combined with enzymatic probing (e.g., RNases) [60,61,62,63]. These approaches are based on the formation of chemical adducts that preferentially modify single-stranded regions, which are then detected through reverse transcription stops and resolved on denaturing gels to generate nucleotide reactivity profiles [64,65,66]. However, these methods present important limitations, including the relatively small difference in reactivity between single- and double-stranded regions, which reduces structural discrimination, and individual reagents typically exhibit base specificity, resulting in incomplete and biased structural information [63,67].

**Figure 2:**
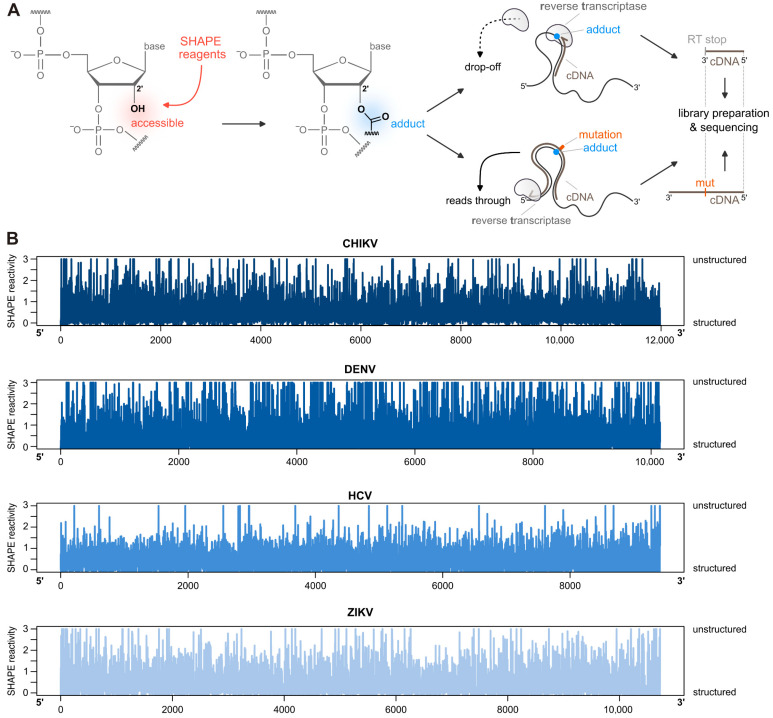
Genome-wide RNA structure profiling using SHAPE reactivity. (**A**) The principle of SHAPE (Selective 2′-Hydroxyl Acylation analyzed by a primer extension). SHAPE reagents selectively react with 2′-hydroxyl residues that reside in flexible regions of an RNA sequence. During reverse transcription, when reverse transcriptase encounters a nucleotide bearing a SHAPE adduct, the enzyme can either stall and dissociate from the RNA–cDNA hybrid, generating truncated cDNA products, or read through the modification and incorporate a mutation at the corresponding position. Mapping these stops or mutations along the RNA sequence allows for the identification of flexible nucleotides and inference of the RNA secondary structure. (**B**) Genome-wide SHAPE reactivity profiles across representative viral RNA genomes, such as the Chikungunya virus (CHIKV), the Dengue virus (DENV), the Zika virus (ZIKV), and the Hepatitis C virus (HCV). Each bar corresponds to the reactivity of an individual nucleotide. High SHAPE reactivity indicates flexible, generally unpaired nucleotides, whereas low reactivity corresponds to structurally constrained or base-paired regions, enabling the identification of structured elements throughout the viral genome.

SHAPE overcomes these limitations by exploiting the intrinsic reactivity of the ribose 2′-hydroxyl group, whose chemical accessibility reflects local nucleotide flexibility (Figure 2A). The 2′-OH is highly reactive in single-stranded or conformationally dynamic regions but becomes constrained upon base pairing. Accordingly, the selective acylation of the 2′-OH generates 2′-O-adducts preferentially at flexible nucleotides [68,69]. These modifications block reverse transcriptase during cDNA synthesis, producing termination patterns that can be used to infer the RNA structure [68,70]. The resulting cDNA fragments are then resolved, typically by capillary electrophoresis, enabling nucleotide-resolution mapping of RNA secondary and tertiary structures [37].

SHAPE’s main output is a reactivity profile of each nucleotide, which is computed based on its binding with the chemical probe. High reactivity characterizes flexible regions composed of nucleotides in a single-stranded conformation. On the other hand, low reactivity, including completely unreactive nucleotides (i.e., reactivity = 0), highlights structured regions composed of double-stranded nucleotides (Figure 2B). Moreover, SHAPE’s reactivities have been used to improve the predictive power of thermodynamic-based algorithms, including RNAfold and RNAstructure [38,71,72]. The reactivity of each nucleotide is employed as a soft constraint during the predicted folding, limiting the degree of freedom of the RNA and consequently improving computational time and predicting power.

The genome of RNA viruses follows the same folding rules as other RNA molecules and exhibits specific structural regions, which are crucial for viral replication and fitness [73,74]. Through the advancement in high-throughput technologies, High-Throughput SHAPE (hSHAPE) was developed to examine the first 900 nucleotides of the HIV-1 genome, which represent an important functional region [42]. This study shed light on the structure of the HIV-1 5′ UTR, showing that this region is highly structured and conserved. Furthermore, this work provided one of the first direct maps of how the viral nucleocapsid (NC) protein interacts with the genomic RNA inside intact virions [42]. Follow-up studies extended the analysis on the whole HIV-1 genome (~9000 nucleotides), uncovering numerous unknown structural elements [29] (Table 1).

With the development of next-generation sequencing (NGS), the SHAPE methodology was updated by adding NGS readout, developing SHAPE-seq, and achieving genome-wide structural information [75,76,77]. Here, to distinguish among the different species of RNA, each molecule is barcoded with a unique nucleotide sequence near the 3′ end. The RNA pool is divided into two populations: a positive pool, which is treated with SHAPE reactives (e.g., 1-methyl-7-nitroisatoic anhydride, also defined as 1M7 [78]), and a negative pool treated with a control solvent. After treating the two RNA populations, the samples are reverse transcribed to generate cDNA libraries whose fragment lengths reflect the presence or absence of chemical modifications that halt reverse transcription (Figure 2A). To add the required Illumina sequencing adapters to the cDNA products, one of the adapters is included in the tail of the RT primers, and the other is added through a single-stranded DNA ligation step after the RNA is removed by NaOH hydrolysis. Finally, the cDNA molecules can go through NGS-based sequencing and be processed with bioinformatic tools [79,80].

An optimized version of SHAPE-seq, termed SHAPE-seq 2.0, was later developed as a more standardized, “kit”-like protocol, applicable to diverse RNA targets [81]. However, it exhibited two main limitations: a reliance on reverse transcription stop counts, which can introduce noise and false positives, and an incompatibility with in vivo measurements. These limitations were addressed by subsequent approaches, including In Vivo Click SHAPE (icSHAPE) [82] and SHAPE mutational profiling (SHAPE-MaP) [43,83].

By introducing the SHAPE reagent NAI-N_3_, which allows efficient RNA modification in vivo, Spitale et al. established the in vivo click-selective 2′-hydroxyl acylation and profiling experiment (icSHAPE) [82]. The development of NAI-N_3_ originated from the chemical modification of an existing in vivo SHAPE reagent, 2-methylnicotinic acid imidazolide (NAI) [78]. By introducing an azide group at position 2 of the nicotinic acid ring, NAI was converted into a dual-function probe capable of both RNA structure probing and subsequent enrichment. NAI-N3 acylates the 2′-OH of RNA, leaving an azide group exposed, allowing the DIBO-biotin specifically to “click” to the nucleotides that have been modified by the SHAPE reagent. The modification blocks the RT enzyme, leading to the detection of single-stranded nucleotides by reverse transcription followed by deep sequencing and bioinformatics analysis [84]. This strategy was recently employed to perform an in vivo structural characterization of the Severe Acute Respiratory Syndrome Coronavirus 2 (SARS-CoV-2) genome in infected cells [47].

The main change in SHAPE-MaP is the interpretation of the SHAPE chemical modifications. In this approach, the RT is not stopped by the presence of SHAPE reagents (NMIA or 1M7) but, instead, it is able to introduce a non-complementary nucleotide where the enzyme finds a SHAPE-modified nucleotide (i.e., insertion of a mutation), creating a mutational profile. This occurs because reverse transcriptase enzymes can, in some cases, read through unusual 2′-O-linkages and adducts, after enzyme pausing [85,86]. Under these conditions, when the enzyme encounters a SHAPE adduct and pauses, the probability of nucleotide misincorporation at that site is substantially increased. The ability of the RT enzyme to read through the adduct is also enhanced by the presence of Mn^2+^, which promotes mutagenic behavior [87]. SHAPE-MaP was successfully employed to profile and characterize the secondary structure of different RNA viruses, including HIV-1 [43], Chikungunya virus (CHIKV) [44], and DENV [7]. SHAPE-Map was further optimized to also be used in vivo [88], which made it possible to investigate viral RNA structures directly inside infected cells [89]. The in vivo approach, in combination with the in vitro one, was recently applied to study the complete secondary structure of the WNV [45], DENV, and ZIKV [46] genomes, revealing their highly structured genome with extensive long-range interactions. Overall, SHAPE methodologies have been used to profile different RNA viruses such as CHIKV [44], DENV [7], ZIKV [46], and HCV [49] (Figure 2B and Table 1).

Collectively, icSHAPE and SHAPE-MaP have helped scientists to take measurements of the RNA structure across entire transcriptomes and full-length RNA molecules at a single nucleotide level [43,82,90] (Table 1). However, they present many differences: (i) the reagents that are used differ in terms of reactivity and solubility; icSHAPE utilizes NAI-N3 instead of 1M7 or NMIA, which are used in SHAPE-MaP. (ii) SHAPE-MaP relies on cDNA mutations, while icSHAPE relies on cDNA truncation. (iii) icSHAPE libraries are only enriched with structurally informative molecules (acetylated ones), while, in SHAPE-MaP, every sequence generates a cDNA.

In vitro and in vivo SHAPE analyses differ in the biological context in which they are applied. The in vitro SHAPE is performed on purified and in vitro-transcribed RNA; in this way, due to the absence of other interactors, the RNA folding is only influenced by thermodynamics and ionic strengths [37]. Instead, regarding in vivo SHAPE, it is carried out in living cells, where RNA exists in a highly dynamic environment. Here, RNA can interact with a wide variety of molecules, such as ribosomes, RNA-binding proteins, and other regulatory factors; consequently, in vivo SHAPE reflects the structural dynamics of RNA in the cellular environment [82]. While in vitro offers cleaner, simpler structural snapshots, in vivo data, though noisier, captures the biologically relevant, dynamic RNA structures in their native context, often revealing protein interactions or unique folding patterns that in vitro experiments could miss [91].

The SHAPE reagents 1M7, 1M6, and NMIA have been extensively validated for the study of RNA structures [43,92,93,94,95] (Table 2). However, the reagents differ in terms of applicability, including resolution, cell-permeability, half-life, and solubility [96]. Furthermore, several upgrades of SHAPE reagents have been proposed, including a longer half-life (i.e., FAI and NAI) [97] or enhancing adduct detection by the addition of biotin molecules coupled with the SHAPE reagent (i.e., N-propanone isatoic anhydride (NPIA) or NAI-N_3_) [82,98] (Table 2).

The development of SHAPE and its high-throughput derivatives has revolutionized the study of viral RNA by enabling the accurate, nucleotide-level structural mapping of large genomes. By overcoming the limitations of earlier probing approaches and allowing both in vitro and in vivo analyses, these methods have provided powerful tools to uncover the structural elements of viral replication, regulation, and pathogenicity using SHAPE.

## 3. Functional Elements in the 5′ Untranslated Region of Viral RNAs

The 5′ UTR is a densely regulated region in RNA viruses, hosting structures essential for viral translation, interaction with host proteins, and the control of viral fitness and virulence [8,100]. Viral 5′ UTRs adopt defined and often conserved architectures that function through modular stem-loops, pseudoknots, tertiary junctions, and long-range interactions.

In ssRNA(+) viruses, 5′ UTRs often regulate translation through IRESs, structured RNA elements that promote cap-independent initiation by directly recruiting ribosomes and associated factors (Figure 1) [101]. Through these RNA elements, ribosomes are recruited directly to the viral RNA, thereby bypassing the canonical 5′ cap-dependent scanning mechanism. This strategy ensures viral protein synthesis even when host cap-dependent translation is suppressed, for example, during antiviral responses. The IRES elements are functionally classified according to their structural organization and dependence on host initiation factors, ranging from more factor-dependent and structurally flexible elements (type I/II) to highly compact RNAs that directly engage the ribosome with minimal auxiliary factors (type III/IV) [102,103].

**Figure 3:**
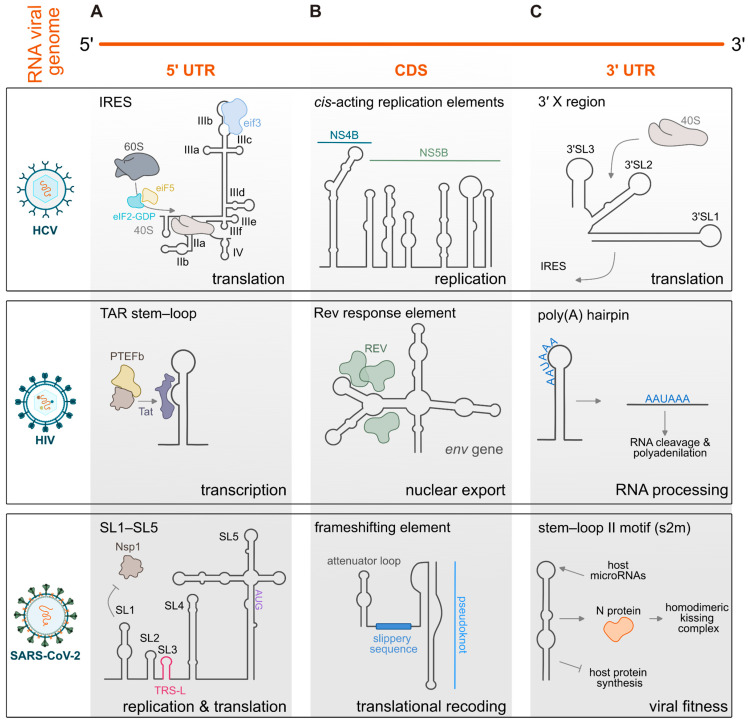
The functional RNA structural elements across viral genomes. A schematic overview of representative structured RNA elements located in the 5′ UTR, CDS, and 3′ UTR of selected RNA viruses, highlighting their roles in translation, replication, RNA processing, nuclear export, translational recoding, and viral fitness. (**A**) Examples of regulatory structures in the 5′ UTR. In HCV, the internal ribosome entry site (IRES) recruits the 40S ribosomal subunit and translation initiation factors, including eIF3, eIF2-GTP, and eIF5, to promote cap-independent translation initiation. In HIV, the TAR stem-loop interacts with Tat and recruits P-TEFb to stimulate transcriptional elongation. In SARS-CoV-2, conserved stem-loops SL1–SL5 contribute to replication and translation, with SL1 counteracting Nsp1-mediated host translation inhibition and SL3 containing the transcription regulatory sequence leader (TRS-L). (**B**) Representative RNA structures embedded within viral coding regions. In HCV, cis-acting replication elements within the NS4B and NS5B coding regions contribute to viral RNA replication. In HIV, the Rev response element (RRE), located within the *env* coding region, binds Rev and mediates the nuclear export of viral RNAs. In SARS-CoV-2, the programmed ribosomal frameshifting element, composed of a slippery sequence and downstream structured RNA, promotes the translational recoding required for production of downstream viral proteins. (**C**) Examples of structured RNA elements in viral 3′ UTRs. In HCV, the 3′ X region contributes to long-range interactions with the IRES and supports translation. In HIV, the poly(A) hairpin regulates the accessibility of the AAUAAA polyadenylation signal and RNA processing. In SARS-CoV-2, the stem-loop II-like motif (s2m) can interact with host cellular microRNAs, including miRNA-1307-3p, modulate host protein synthesis through structural mimicry, and can form a homodimeric kissing loop complex assisted by the viral nucleocapsid protein, suggesting roles in host–virus interactions and viral genome organization.

The Hepatitis C virus (HCV) IRES represents one of the most extensively characterized examples and belongs to the type III class. It comprises three major structural domains (II–IV) organized into a compact and modular architecture. Unlike canonical mRNAs, the HCV IRES directly recruits the 40S ribosomal subunit to the vicinity of the AUG start codon without the need for scanning [104,105]. Following 40S binding, the IRES–ribosome complex associates with eIF3 and the ternary Met-tRNAi-eIF2-GTP complex to assemble a translation pre-initiation complex (Figure 3A). Domains II and III contain extended stem-loop structures with multiple subdomain loops that establish direct contact with the 40S subunit (Figure 3A). The basal region of domain III folds into a conserved pseudoknot that spatially organizes the RNA to correctly position the AUG codon [106]. The central pseudoknot domain acts as an architectural hub, connecting the start codon, containing domain IV, with the other major domains and ensuring the correct positioning of the 40S subunit.

These structured modules are connected by flexible hinge regions that allow conformational adaptability [48,107,108]. Importantly, the HCV IRES does not adopt a single rigid conformation but is better described as an ensemble of conformers [109]. Consistent with this duality, SHAPE analyses confirmed the strong structural constraints within the IRES region, in contrast to the pseudoknot-stem termini that are more flexible. SHAPE, in the presence of saturating 40S subunits, revealed decreased reactivity across the IIIe stem-loop, consistent with ribosome engagement, while nucleotides surrounding the AUG exhibited increased flexibility, reflecting the unfolding of domain IV to permit translation initiation [48,49]. Genome-wide SHAPE-MaP further demonstrated that the HCV IRES is under strong evolutionary constraint across genotypes [49].

Similarly, the IRES of FMDV is organized into four major structural domains (II–V). Domain III plays a central role in ribosome recruitment, whereas domain IV interacts with eIF4G through a characteristic Y-shaped three-way junction [103]. Structural probing analyses comparing in vitro and in vivo SHAPE reactivity profiles revealed differences in the protection patterns of both the IRES element and the 3′ UTR, indicating that the RNA adopts distinct conformations in the cellular cytoplasm compared to the naked RNA probed in vitro. In particular, specific residues located in the apical region of domain III were protected in cells but appeared flexible in vitro, consistent with RNA–protein or RNA–RNA interactions occurring in the cytoplasmic environment [50]. Covariation analysis and conserved helix prediction supported this structural plasticity, providing evidence for dynamic long-range interactions between these distant regions and highlighting a functional crosstalk that contributes to efficient IRES-driven translation in cells [50].

A comparable level of structural and functional complexity is observed in the 5′ UTR of Enterovirus 71 (EV71), a member of the Picornaviridae family. This region comprises six stem-loop structures (SLI–SLVI), with SLII–SLVI forming the IRES. Within this architecture, SLII contains a characteristic bulge that mediates interactions with host RNA-binding proteins, including hnRNP A1, which enhances IRES-mediated translation, and AUF1, which competes for the same site and suppresses translation, thereby providing a mechanism for fine-tuning translational output [110,111,112].

Although IRES-mediated translation is one of the best-characterized functions of viral 5′ UTRs, these regions also contain structurally diverse cis-acting elements that regulate additional stages of the viral life cycle. One of the most extensively characterized examples is the HIV-1 5′ UTR. The first genome-wide structural analysis of the HIV RNA using SHAPE identified multiple regions with low reactivity and high base-pairing probability, including the 5′ UTR and the Rev responsive element (RRE) [29]. The 5′ UTR has subsequently been extensively investigated by chemical probing approaches, which defined a series of conserved structural elements [43,113,114,115,116].

The HIV 5′ UTR is rich in cis-acting elements crucial for the Tat-mediated activation of viral transcription, reverse transcription, and genome packaging. These are the trans-activation response (TAR) element, the poly(A) hairpin, the U5-PBS complex, and stem-loops 1–4 containing the dimerization site DIS, the major splice donor, and the major packaging signal [117]. The TAR stem-loop, characterized by a 3-nt UCU bulge, is recognized by the viral Tat protein. Tat binding promotes the recruitment of the positive transcription elongation factor b (P-TEFb) and the assembly of the super elongation complex (SEC), thereby enabling efficient transcription of the full-length viral genome [108,118] (Figure 3A). The DIS element, located within stem-loop 1, consists of a conserved palindromic loop forming a stable hairpin structure. The self-complementary loop sequence enables kissing loop interactions between two RNA monomers through loop–loop base pairing [119,120,121]. Dimerization is a key step in the HIV-1 life cycle, as the retroviruses package two copies of their genome into viral particles. These genomes are non-covalently associated through the DIS motifs. The structures of both monomeric and dimeric RNA conformations have been resolved using an approach that integrates RNA structural probing with high-throughput functional profiling (i.e., FARS-seq) [120]. These elements illustrate how local RNA modules act as dynamic switches controlling multiple stages of the retroviral life cycle [122,123,124].

In flaviviruses, 5′ UTRs tend to be structurally conserved despite variable sequences and are essential for viral RNA synthesis, containing multiple specific structural elements that regulate replication and translation [125]. A large Y-shaped stem-loop of ~70 nucleotides, termed stem-loop A (SLA), is required for the binding of the viral RNA-dependent RNA polymerase NS5 and acts as a promoter for viral RNA synthesis [126] (Figure 1). SLA-mediated RNA replication is a conserved mechanism across flaviviruses, and its structure is tightly linked to viral fitness; deletion or disruptive mutations of SLA essentially abrogate viral replication [127]. The SLA domain is often followed by a smaller SLB domain, which functions in tandem with SLA as a core 5′-UTR promoter module; similar SLA–SLB arrangements have been identified in different flaviviruses, including Yellow fever virus (YFV) [128] and West Nile virus (WNV) [45]. In DENV, SLA and SLB are separated by an oligo(U) linker sequence that contributes to their proper spatial and functional arrangement during replication [45].

SHAPE-MaP experiments identified different functional structural elements in the 5′ UTR of the alphavirus CHIKV, which include stem-loop 3 and 4 (SL3, SL4) [44]. Interestingly, the SL3 structure is the crucial element for the domain functionality, acting together with the structural element of the 5′ CSE, and is essential for viral replication [44,129].

Other viral families, such as betacoronaviruses, also rely on highly structured 5′ UTRs composed of multiple functional stem-loops [130]. For example, SARS-CoV-2 5′ UTR contains five different functional stem-loops (SL1–SL5) [131] (Figure 3A). SL1 within the 5′ leader region counteracts Nsp1-mediated host translation inhibition, thereby enabling selective translation of viral RNAs [132,133] (Figure 3A). SL2 adopts a conserved tetraloop structure and has been implicated in viral replication [131,134]. SL3 contains the transcription regulatory sequence leader (TRS-L), which is essential for discontinuous transcription and subgenomic RNA synthesis [135]. SL4 forms a relatively long and stable hairpin involved in regulating subgenomic RNA production, whereas SL5 comprises a more complex domain organized around a four-way junction.

Together, these studies demonstrate that the 5′ UTR constitutes a structurally encoded regulatory hub in viral genomes, where conserved and dynamic RNA architectures orchestrate translation initiation, replication, genome packaging, and adaptation to host cellular environments across diverse RNA virus families.

## 4. Structural Elements in the Coding Regions of Viral RNAs

Viral RNA secondary structures are not confined to UTRs but are also embedded within CDSs, where they can exert important regulatory functions, often associated with or including protein-binding domains. Among these structural elements, some are known to promote programmed ribosomal frameshifting, thereby increasing the coding capacity of the viral genome by shifting into an alternative reading frame [5] (Figure 1).

A prominent example of extensive structural regulation within coding regions is provided by HCV, whose CDS harbors a complex network of regulatory RNA structures. [54] (Figure 3B). Through SHAPE profiling, mutagenesis analyses, and functional validation, these structures have been characterized as conserved but functionally diverse, involved in several steps of the viral life cycle. For instance, conformational changes in the RNA structure SL6038 regulate replication, while the kissing interaction between the SL427 and SL588 structures is important for infectivity [54]. Structured RNA elements within the central region of the HCV genome contribute to immune evasion by shielding sequences that would otherwise be cleaved by RNase L [49]. Searches for *cis*-acting replication elements (CREs) identified a marked enrichment of structured RNA motifs within the NS4B and NS5B coding region, that modulate the efficiency of viral replication [136] (Figure 3B).

Similar coding-region replication elements have also been described in picornaviruses. A genome-wide, single-nucleotide resolution RNA structure map of PV identified multiple conserved structural elements within the open reading frame, including a functionally essential RNA structure in the 3D^pol^ coding region whose disruption impairs replication and infectivity [40].

Regulatory RNA structures within CDSs have also been extensively described in retroviruses such as HIV. Pseudoknots located downstream of slippery sequences modulate the abundance of the *gag–pro* or *gag–pol* genes in HIV [137]. Stable stem-loop structures, identified in HIV-1 and HIV-2, have likewise been shown to possess frameshift-promoting activity [138,139]. In HIV, the RRE, composed of a series of stem structures within the envelope (*env*) coding region, is recognized by the Rev protein, which is essential for the translocation of viral RNA from the nucleus to the cytoplasm through the nuclear pore complex [140,141] (Figure 3B). In addition, SHAPE-based analysis has revealed RNA secondary structures within HIV CDSs, often positioned in proximity of autonomously folding protein domains [29]. The observed correlation between the RNA secondary structure within CDS and the structural organization of the encoded proteins suggests that such structures may slow ribosome elongation, creating temporal windows that facilitate co-translational folding of the nascent polypeptide.

Coding-region RNA structures are particularly abundant and functionally diverse in flaviviruses. In their capsid-coding region, flaviviruses contain *cis*-acting elements that regulate genome cyclization [55,56,57,142,143]. Smaller functional structures in the flavivirus 5′ region include the capsid-coding region hairpin element (cHP) and the 5′ conserved sequence element (CSE) [129,142]. cHP lies in the very beginning of the coding region, an element potentially crucial for initial codon recognition and initiation factor recruitment [142]. Mutations in the cHP of DENV and WNV inhibit viral replication [142]. The CSE is a small conserved region essential to circularize the viral genome by interacting with its counterpart in the 3′ and forming a specific panhandled structure [144,145]. The 5′ CSE is an important element also in CHIKV, affecting viral replication and fitness when mutated [44].

Beyond these conserved cyclization signals, four highly structured RNA elements were mapped within the WNV coding region, and disruption of these structures using antisense LNA (Locked nucleic acid) oligonucleotides resulted in impaired viral replication in cell-based infection assays [45].

Regulatory structures across the coding sequence were also found in four different dengue serotypes [7]. Boerneke et al. applied SHAPE-MaP to identify regulatory motifs using a structure-first approach, rather than starting from sequence conservation, allowing the identification of previously undiscovered motifs. They identified at least ten motifs within the coding sequence, often interacting with proteins, which can impact viral fitness. These RNA motifs promote a compact global genome architecture and regulate the DENV replication cycle.

In addition to the local structural elements, flaviviral genomes also engage in long-range RNA–RNA interactions that contribute to genome organization and viral fitness. For example, combined icSHAPE and psoralen analysis of RNA interactions and structures (PARIS, method used for RNA–RNA interaction identification) identified extended interactions in ZIKV Asian strains, including a functionally relevant interaction between the 5′ UTR and the E protein coding region that is important for viral infectivity [58]. The analyses of Influenza A virus (IAV) coding regions have identified conserved RNA secondary structure motifs that persist despite sequence variation [146]. Conserved and functionally essential RNA secondary structures were systematically identified by combining SHAPE probing with genetic and functional validation [59], leading to the identification of packaging stem-loop 2 (PSL2), a highly conserved structural element within the coding region that is required for genome packaging and preserved across all known IAV isolates.

Another important role of coding-region RNA structures is the regulation of translational recoding, a mechanism prominently exploited by coronaviruses, essential for the production of the viral RNA-dependent RNA polymerase (RdRp/Nsp12) and downstream nonstructural proteins [146]. Frameshifting is triggered when the translating ribosome encounters structured RNA elements, most commonly pseudoknots, which promote a shift in the reading frame [59] (Figure 3B). However, alternative RNA conformations can also contribute, as long-range interactions such as kissing loop–loop contacts have been shown to enhance frameshifting efficiency and influence the balance between full-length and subgenomic viral RNAs [143].

Beyond controlling viral protein synthesis, coronavirus RNAs can also establish direct structural interactions with host transcripts. For instance, SARS-CoV-2 RNA can form base-pairing interactions with the 3′ UTRs of host mRNAs, leading to their stabilization through the recruitment of the RNA-binding protein YBX3, which results in immunopathogenic effects [147]. This finding highlights how viral RNA function is not solely dictated by its intrinsic sequence and structure but can be modulated by the cellular environment, including interactions with host RNAs.

Together, these examples underscore that RNA structures embedded within viral coding sequences provide an additional regulatory layer that coordinates translation, replication, and host interactions beyond their protein-coding function.

## 5. Structural Elements in the 3′ Untranslated Region of Viral RNAs

Beyond coding regions and 5′ UTR elements, the 3′ UTR of many RNA viruses contains highly structured regulatory motifs that coordinate genome stability, translation, replication, and interactions with host factors. The 3′ UTR in HIV-1 is characterized by highly structured and conserved regulatory regions [29,148]. SHAPE analysis has revealed that the 3′ UTR of the *Nef* gene, encoding for the negative factor protein, contains multiple secondary structure elements, with low SHAPE reactivity and high pairing probability [29]. This region has been demonstrated to be involved in HIV-1 replication by showing that, when targeted by multiple host miRNAs, specifically miR-29a/b and miR-223, there is a downregulation of HIV-1 replication [149]. Downstream of the *Nef* coding region, the HIV-1 3′ terminal region contains a polyadenylation (poly(A)) hairpin signal. Although the AAUAAA motif is present at both the 5′ and 3′ ends of HIV-1 RNA, polyadenylation normally occurs at the 3′ end, whereas usage of the 5′ signal is suppressed. It has been proposed that the local RNA structure contributes to this differential regulation by modulating accessibility of the poly(A) signal [150] (Figure 3C).

In HCV, although the IRES is located in the 5′ UTR, the 3′ UTR also contributes to IRES-dependent translation through interactions with the 40S ribosomal subunit [51,151]. Following recruitment, the 40S subunit can be repositioned onto the IRES (Figure 3C). Moreover, the NS5B coding region upstream of the HCV 3′ UTR engages in long-range kissing interactions with IRES domain IIId and the 3′X region—a highly structured 98-nucleotide element within the 3′ UTR [152]—indicating close spatial proximity between the 5′ and 3′ ends of the genome [153,154]. In addition, several host and viral proteins involved in HCV translation, including PTB, IGF2BP1, PCBP2, and the core protein, bind both UTRs, promoting RNA circularization and facilitating the efficient recycling of the 40S subunit and eIF3 for subsequent rounds of translation [155,156]. A specific stem-loop (3′SL) in flaviviruses contains Conserved Sequence 1 (CS1), within which an 8-nt sequence (3′CYC) mediates genome cyclization through complementary base pairing with the 5′CYC located in the capsid coding region. This interaction is essential for viral RNA replication in several flaviviruses, including DENV, WNV, and Tick-Borne Encephalitis virus (TBEV), and depends on both sequence and structural determinants [157,158,159,160].

The 3′ UTR of flaviviruses is substantially longer than the 5′ UTR (~400–600 nt) and contains multiple structured domains involved in viral genome packaging, replication, and translation [161]. Beyond their roles in translation and genome cyclization, flaviviral 3′ UTRs also contain specialized RNA structures that directly modulate host–virus interactions. Among these, xrRNA is a conserved and complex structural element present in mosquito-borne flaviviruses that stalls the host 5′–3′ exoribonuclease XRN1, thereby preventing RNA degradation and promoting the accumulation of subgenomic flaviviral RNAs (sfRNAs) within infected cells [15,16] (Figure 1). First discovered in the Murray Valley virus, the xrRNAs were characterized in the majority of mosquito-borne flaviviruses, including DENV, ZIKV, WNV, and YFV [11,52,162]. The production of sfRNAs is linked to different anti-host defense mechanisms, including the interaction with host proteins in order to hijack or disrupt cellular mechanisms, such as the interferon response [14,163]. xrRNAs are often present in tandem (xrRNA1 and xrRNA2) to increase their effect, with the notable exception of DENV4, where only xrRNA2 is present [164,165]. Other functional regions are present downstream of the xrRNA, including the conserved short direct repeats (DRs) and repeated conserved sequences (RCS). Although their biological function is mostly unknown, some specific conserved sequences, including DRs RCS3, CS3, RCS2, and CS2, are associated with all of the known duplicated SL-based and dumbbell (DB)-based stalling structures and are involved in the production of sfRNAs species within infected cells, representing novel candidates for sfRNA formation [166]. DB elements are structural motifs that form pseudoknots and are located in highly conserved regions in the 3′ of MbFVs genomes, including DNV, ZKV, WNV, and YFV [167]. Their role in regulating viral protein translation and genome replication has been demonstrated through studies using mutant viral strains in which mutations disrupting pseudoknot base pairing were introduced [160,167]. Moreover, further evidence supporting their functionality comes from both their location and their interaction with other elements of the 3′ UTR. In particular, the DBs are adjacent to the 3′CS, and in many strains of MbFVS, the pseudoknot can overlap with the 3′CS. This led to the hypothesis that the DB pseudoknot could be used as a sensor for the 5′–3′ base pairing, or as a competitor for the cyclization of the genome, to maximize viral protein production [168].

The functional importance of structured 3′ UTR elements is not limited to flaviviruses but extends to other arboviruses, including alphaviruses. In the alphavirus CHIKV, the Y-shaped stem-loop (SLY) is a highly conserved RNA secondary structure located in the 3′ UTR that enhances viral replication. Notably, its function is host-specific, as it promotes replication in mosquitoes but not in human cell lines. Mutational analyses have shown that replication efficiency depends on the preservation of the SLY secondary structure rather than its exact nucleotide sequence [169,170]. Similar Y-shaped RNA elements have also been predicted in the 3′ UTRs of related alphaviruses, such as the Sindbis virus (SINV), suggesting that this structure represents a conserved feature within the group [170].

Similarly, coronaviruses also rely on conserved 3′ UTR RNA structures to support replication and regulate genome function. The stem-loop II-like motif (s2m) is a highly conserved RNA element located at the 3′ end of several ssRNA(+) viruses, including members of the *Astroviridae*, *Caliciviridae*, *Picornaviridae*, and *Coronaviridae* families (Figure 3C). Its conservation across divergent viral lineages indicates that the motif has been maintained under strong selective pressure, suggesting an important biological function [171,172]. Structural studies further proposed that s2m RNA may act through macromolecular mimicry of a ribosomal RNA fold, potentially allowing it to bind proteins with oligomer-binding-like folds and contribute to the viral hijacking of host protein synthesis [173]. More recently, analysis of the SARS-CoV-2 s2m element showed that it can dimerize through an intermediate homodimeric kissing loop complex, which is subsequently converted into a more stable duplex structure with the assistance of the viral nucleocapsid protein [174]. The same study also identified binding sites within the SARS-CoV-2 s2m element for the host miRNA-1307-3p, which has the potential to regulate the production of several interleukins (Figure 3C). Together, these observations suggest that s2m may contribute not only to viral genome dimerization but also to host–virus regulatory interactions involving cellular miRNAs [174] (Figure 3C). Due to its high conservation and absence in human genomes, s2m represents a potential target for antiviral strategies [173]. Lastly, the hypervariable region (HVR) is a small but conserved sequence contained in the 3′ UTR. Interestingly, HVR exhibits the highest variability when comparing in vitro and in vivo SHAPE data, with a high SHAPE reactivity in vivo [130]. The dynamicity of this region suggests that in vivo HVR could fold into multiple mutually exclusive structures [130].

Together, these examples highlight the 3′ UTR as a multifunctional structural hub whose conserved RNA architectures are essential for viral fitness and represent promising targets for future antiviral strategies.

## 6. Targeting RNA Structures for Therapeutic Purposes

**Figure 4:**
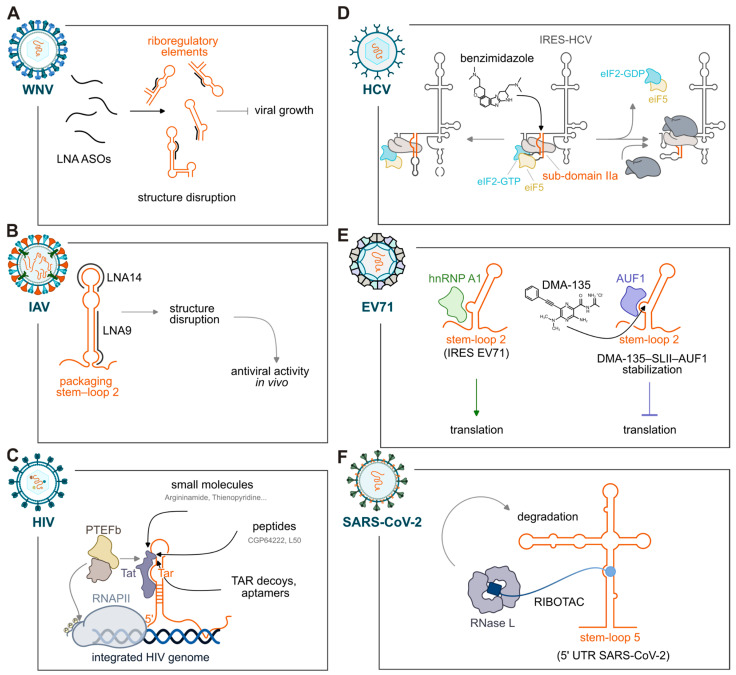
Targeting structured viral RNA elements for antiviral intervention. (**A**) Structured elements within the West Nile virus (WNV) 3′ UTR can be targeted by locked nucleic acid (LNA) antisense oligonucleotides (ASOs), which disrupt RNA structures and inhibit viral growth. (**B**) In the Influenza A virus (IAV), packaging stem-loop 2 (PSL2) is a conserved RNA element required for genome packaging. LNAs targeting PSL2 destabilize the RNA structure and show potent antiviral activity in vivo. (**C**) In HIV, the transactivation response (TAR) element forms a stem-loop that recruits the viral Tat protein and host P-TEFb to activate transcription from the integrated viral genome. Small molecules, peptides, and TAR decoys or aptamers have been developed to disrupt the Tat–TAR interaction. (**D**) The Hepatitis C virus (HCV) internal ribosome entry site (IRES) in the 5′ UTR mediates cap-independent translation. Small molecules, such as benzimidazoles, bind structural motifs within the IRES (for example, subdomain IIa), inducing conformational changes that disrupt ribosome recruitment. (**E**) In EV71, stem-loop II of the IRES interacts with host RNA-binding proteins that regulate translation. Small molecules such as DMA-135 remodel the RNA structure to favor the binding of the inhibitory factor AUF1 over hnRNP A1, thereby repressing viral translation. (**F**) In SARS-CoV-2, RNA-targeting chimeras (RIBOTACs) bind structured elements in the 5′ UTR and recruit the cellular endoribonuclease RNase L, triggering the targeted degradation of viral RNA.

Over the past years, it has become clear that not only proteins but also RNAs can serve as therapeutic targets—despite long being considered “undruggable.” The first evidence that RNA could be pharmacologically targeted came from the discovery of antibiotics such as actinomycin, streptomycin, and neomycin, which bind ribosomal RNAs and block protein synthesis [175,176]. Approaches to therapeutically modulate RNA include antisense oligonucleotides (ASOs) and small molecules as well as small interfering RNAs (siRNAs), CRISPR-based gene editing, and bifunctional molecules [177,178]. Together, these approaches underscore the importance of understanding not only the sequence but also the structural and functional organization of viral RNAs. For ASO-based strategies, detailed knowledge of RNA secondary structure is essential for identifying accessible regions for efficient hybridization, as well as critical structural elements whose disruption can interfere with essential RNA–protein or RNA–RNA interactions. Similarly, the rational design of RNA-targeted small molecules relies on mapping defined structural motifs that can accommodate selective ligand binding. Targeting RNAs offers an additional advantage because functional RNA structures are often more evolutionarily conserved than their primary sequences [179,180]. This allows for a more stable target, which reduces the likelihood of resistance emerging. Stable, conserved, and functionally indispensable RNA elements thus represent attractive and promising targets for the development of RNA-targeted interventions. Building on this concept, recent work has applied SHAPE-MaP to systematically classify structural features across viral genomes by integrating SHAPE reactivity profiles with Shannon entropy measurements. This combined analysis enables the identification of conserved and highly accessible RNA elements that may serve as promising therapeutic targets [179] (Figure 4), with a focus on their application to viral genomes.

## 7. Antisense Oligonucleotides and Related Oligonucleotide-Based Strategies

ASOs are short single-stranded nucleic acid polymers, typically 18–30 nucleotides in length, incorporating diverse chemical modifications to enhance their stability, resistance to nucleases, and cellular uptake. ASOs modulate gene expression through two principal mechanisms. The first relies on Watson–Crick base pairing between the ASO and its target RNA, forming an RNA–DNA heteroduplex that is recognized by RNase H, which selectively degrades the RNA strand, thereby silencing the target transcript. The second is based on a steric-blocking mechanism that does not involve RNase H. In this case, the ASO binds its target and physically occludes protein- or RNA-binding sites, modulating, for instance, translation or the recruitment of regulatory factors [181]. Vitravene (fomivirsen), a 21-mer phosphorothioate 2′-deoxynucleotide ASO, was the first FDA-approved antisense therapeutic and remains the only antiviral oligonucleotide drug to have reached the market. It targets a complementary sequence within the major immediate-early region (IE2) of Cytomegalovirus (CMV), and its primary mechanism of action is RNase H-mediated degradation of the viral mRNA [182,183,184]. Another prominent example is Bepirovirsen, a chemically modified ASO targeting the Hepatitis B virus (HBV). Bepirovirsen promotes the RNase H-mediated degradation of the HBV surface antigen (HBsAg) transcripts and exerts immunostimulatory activity through toll-like receptor 8. The compound has demonstrated encouraging antiviral effects in clinical studies [185,186]. It is currently under evaluation in late-stage clinical trials, and the U.S. FDA has recently granted it a fast-track designation for the treatment of chronic HBV infection [187].

In the antiviral field, ASO-based approaches have been explored against several structured viral RNAs. In this context, 2′-deoxy-2′-fluoroarabinonucleotide (FANA)-modified ASOs represent a particularly versatile platform [188]. FANA is a DNA-mimicking oligonucleotide analog that forms FANA:RNA hybrids resembling DNA:RNA duplexes, thereby enabling efficient recruitment of RNase H1 and cleavage of the target RNA. By directing FANA ASOs to highly conserved and structurally important regions of the HIV-1 genome—including U5, *tat/rev*, and the DIS—the study demonstrated robust antiviral activity. Mechanistic analyses revealed a dual mode of action: RNase H1-mediated degradation of viral RNA (U5 and tat/rev) and a steric hindrance of RNA–RNA interactions essential for genome dimerization at the DIS [188]. Together, these findings underscore the therapeutic potential of phosphorothioate-modified FANA ASOs as a complementary strategy to target structured elements within the HIV-1 RNA genome.

HCV has also been extensively targeted through antisense strategies. Alongside small molecules, ASOs, aptamers, and peptides have also been developed to block HCV IRES activity [189]. The subdomain IIId and domain IV loops constitute particularly responsive antisense targets; notably, a 20-nt phosphorothioate oligodeoxynucleotide targeting the start codon region in domain IV progressed to Phase I clinical testing [190]. Aptamers against subdomain IIId similarly reduced IRES activity in vitro [191,192].

DENV has similarly been targeted at the RNA level using antisense strategies directed against essential structural elements within the genome. Early studies demonstrated that ASOs complementary to the 5′ stem-loop or to the 3′ cyclization sequence could effectively inhibit DENV replication [193,194,195]. Phosphorodiamidate morpholino oligomers (PPMOs), conjugated to arginine-rich peptides to enhance cellular uptake, were shown to suppress DENV-2 replication [193,194]. More recently, vivo-morpholinos (vivo-MOs)—morpholino oligomers conjugated to a dendrimer moiety to facilitate intracellular delivery—have been employed to target conserved structural motifs at the top of the 3′ stem-loop in the DENV 3′ UTR. These vivo-MOs inhibited DENV RNA accumulation, protein expression, and virion production in infected monocyte-derived dendritic cells [193,196]. A more systematic design of vivo-MO sequences targeting multiple conserved genomic regions further revealed that the 3′ UTR constitutes the most effective site for antisense-mediated inhibition of viral replication [197]. Collectively, these studies highlight the potential of ASOs and morpholino-based chemistries to modulate structured RNA elements critical for DENV replication.

As we previously described, UTRs often contain highly conserved regulatory elements that make them attractive targets for antiviral drug development. EV71 has also been targeted through several complementary RNA-based therapeutic strategies. Multiple siRNAs have been designed to silence viral gene expression effectively [198]. In addition, an ASO directed against the structured 5′ UTR—designed based on predicted mRNA secondary structure—demonstrated significant antiviral activity by blocking this essential regulatory element [199]. The 3′ UTR of WNV contains well-characterized structural elements that contribute to immune evasion and pathogenicity by generating sfRNA, a mechanism conserved among flaviviruses. Using SHAPE-MaP to determine the complete secondary structure of the WNV genome in both arthropod and mammalian cells, Huston et al. uncovered a tripartite organization at the 3′ end, with a flexible single-stranded segment (Domain II) flanked by two highly structured regions (Domains I and III) [45]. By combining structural mapping with antisense ASO LNA perturbations, the study showed that several of these structured motifs are essential for viral fitness and that their perturbation causes viral growth defects [45] (Figure 3A). These findings highlight conserved RNA features that are amenable to antisense targeting and suggest that structural conservation across strains can guide the identification of functional RNA elements in other vector-borne viruses.

CHIKV is an important arthropod-borne, ssRNA(+) virus for which no licensed vaccines or antiviral therapeutics are currently available. Building on the SHAPE-based profiling of the 5′ UTR, Prosser et al. designed LNA ASOs targeting RREs within the 5′ region of the genome [200]. These LNAs were optimized to anneal to and destabilize the formation of crucial RNA structures, thereby blocking their interaction with necessary trans-acting factors. Several candidates exhibited strong antiviral activity, underscoring the promise of targeting conserved structural motifs as a strategy for CHIKV inhibition.

RNA secondary structure motifs that persist despite sequence variation [146] have been identified in IAV. These stable structural features represent attractive targets for oligonucleotide- or small molecule-based antiviral strategies. In this context, structure-guided approaches have enabled the identification of conserved and functionally essential RNA elements, such as the packaging stem-loop 2 (PSL2), which is required for genome packaging and preserved across IAV isolates [59] (Figure 4B). Notably, targeting PSL2 with LNAs conferred complete protection in a lethal infection model, highlighting the remarkable therapeutic potential of RNA structure-based targeting strategies and the importance of incorporating structural information into ASO design [201]. SARS-CoV-2—the causative agent of COVID-19—has been responsible for the most extensive respiratory virus pandemic since the 1918 influenza outbreak [202]. In parallel with vaccine development, substantial efforts have focused on RNA-targeted antiviral strategies, including ASOs, aptamers, siRNAs, and RIBOTACs, advancing the field of RNA-targeted drugs. Complementary RNA-targeted approaches have also been applied to the SL1, SL3, and broader 5′-end regions of the SARS-CoV-2 genome. ASOs, including LNAs and peptide-conjugated morpholino oligomers (PPMOs), have been used to interfere with these structured elements and disrupt viral translation [203,204]. Notably, an LNA ASO targeting the 5′ leader sequence, 5′-ASO#26, efficiently disrupted the conserved SL1 stem-loop and blocked viral replication in human cells at nanomolar concentrations. DMS-MaPseq analysis in infected Huh-7 cells confirmed the dose-dependent destabilization of SL1, demonstrating that direct interference with essential 5′ UTR structures can robustly impair SARS-CoV-2 replication [204].

## 8. Small Molecules Targeting Structured Viral RNAs

Besides ASOs, small molecules—traditionally developed to target proteins—have increasingly emerged as modulators of RNA structure and function. The growing recognition of the RNA diverse structural landscape has driven efforts to identify drug-like compounds capable of binding specific RNA motifs and altering their regulatory roles. RNA adopts complex secondary and tertiary structures, creating unique three-dimensional folds, bulges, and grooves that can act as selective and druggable pockets [205,206].

Non-coding regions of RNA viruses have been extensively exploited as drug targets, with HIV representing a prominent example [207,208,209]. HIV replication relies on two essential structured RNA elements: the TAR element, which binds the Tat protein to activate transcription from the long terminal repeat promoter, and the RRE, which recruits the accessory protein Rev to promote nucleocytoplasmic export of viral RNA. The activity of TAR is critically dependent on its stem-loop secondary structure, prompting efforts to develop molecules that interfere with the Tat–TAR interaction (Figure 4C). Early high-throughput screening focused on identifying small molecules capable of disrupting this interaction [210,211]. Argininamide was subsequently shown to inhibit TAR function by locking the RNA into a single conformation [212]. Additional screenings identified small molecules such as Thienopyridine that stabilizes the TAR hairpin [213], amiloride-based ligands targeting the TAR apical loop that effectively blocked Tat binding [207,214], and a hybrid peptoid/peptide oligomer was found to disrupt the Tat–TAR complex [215]. More recently, a virtual screening across a diverse ensemble of TAR conformations identified six TAR-interacting compounds, one of which inhibited long terminal repeat promoter activation by 81% in T-cell lines [216].

Similarly, the RRE has been explored as a target for small molecules aimed at blocking Rev binding [217,218,219,220]. For instance, diphenylfurans bind the RRE hairpin and induce a conformational change essential for inhibiting the formation of the Rev–RRE complex [217,219]. Screening efforts have also identified peptides with higher affinity for RRE than Rev itself, providing an additional route to disrupt Rev function [219].

HCV IRES located in the 5′ UTR is one of the best-characterized viral RNA structures and is essential for cap-independent translation [221,222]. Owing to its defined architecture and key role in infection, the IRES has been widely exploited as a therapeutic target, leading to numerous screening efforts that identified small molecules capable of binding the IRES and blocking translation [222,223,224]. Within the IRES, subdomain IIa acts as an RNA switch, and ligands that stabilize specific IIa conformations can suppress translation [189]. Optimized benzimidazoles are among the most advanced examples; they bind the IIa domain, induce conformational changes that disrupt ribosome–IRES interactions, and show robust activity in replicon assays (Figure 4D) [225,226,227,228]. A FRET-based screen further identified a benzoxazole scaffold that binds IIa and inhibits IRES-mediated translation [229]. A comparable approach has been used to target the IRES of FMDV. Benzimidazole-based small molecules were identified as inhibitors of FMDV IRES function, and SHAPE probing showed that one compound, IRAB, increased local structural flexibility within domain 3, altering three stem-loop elements [230]. The findings indicate that IRAB engages a folded RNA motif spanning the apical region of domain 3 and induces conformational changes in a portion of the IRES that is essential for its activity. Beyond the 5′ UTR, high-throughput screening across the full HCV genome identified small molecules that bind conserved stem-loop motifs within the 3′ UTR—structures critical for replication and genome circularization—and effectively block viral replication [231].

A comparable structure-based strategy has also been pursued in flaviviruses and picornaviruses. In EV71, small molecule screening approaches have identified ligands targeting defined structural elements within the 5′ UTR, particularly the SLII domain [232,233]. Among these, the amiloride-derived compound DMA-135 binds the SLII bulge and induces a conformational rearrangement of the RNA that modulates its interaction with host factors. Specifically, DMA-135 stabilizes the association of the translational repressor AUF1 while disfavoring binding of hnRNP A1, thereby shifting the regulatory equilibrium toward translation repression and resulting in antiviral activity in cell culture (Figure 4E) [232]. Small molecule approaches have also been explored to inhibit WNV replication. A screening strategy aimed at identifying antiviral compounds effective against WNV and related flaviviruses highlighted a class of secondary sulfonamides. Among these, the compound AP30451 emerged as a selective inhibitor of flaviviral RNA translation and was shown to efficiently suppress WNV replication across multiple cell types [234].

SARS-CoV-2 has likewise been explored through structure-directed small molecule approaches. A notable study identified amiloride-derived compounds capable of inhibiting replication of both OC43 and SARS-CoV-2 by binding structured elements in the 5′ genomic region [235]. An initial screen in OC43-infected Vero E6 cells yielded three lead molecules—DMA-132, DMA-135, and DMA-155—and subsequent NMR analyses showed that these compounds preferentially interact with stem-loop structures containing prominent internal or bulge loops. Importantly, these small molecules reduced SARS-CoV-2 replication and represent the first antivirals shown to directly target conserved RNA stem-loops at the 5′ end of the coronavirus genome [235]. This work provides a compelling framework for the development of RNA-directed therapeutics that may be broadly applicable to future coronavirus outbreaks. Additional work has aimed at disrupting SL1, a key structural element at the 5′ end of the SARS-CoV-2 genome that contributes to the enhanced translation of viral transcripts. Using NMR-based screening of a broad fragment collection. Toews et al. identified several small molecules capable of binding this motif. The most promising candidates were subsequently examined in a cell-free translation system, where two compounds—A.2 and A.13—displayed marked and selective suppression of SL1-mediated translation [236].

Beyond stem-loop elements, more complex RNA structures, such as G-quadruplexes (G4s), have also been explored as antiviral targets [178,237,238]. G4s are four-stranded structures stabilized by stacked guanine tetrads, and putative RNA G4 motifs are conserved across betacoronaviruses, including SARS-CoV-2, suggesting important roles in replication and gene regulation. One study focused on an RNA element (RGQ-1) within the SARS-CoV-2 nucleocapsid gene that can adopt either a hairpin or a G4 fold [239]. Tetraphenylethene (TPE) derivatives were designed to shift this equilibrium toward the G4 conformation, resulting in reduced nucleocapsid protein expression and decreased viral levels in Vero E6 and A549 cells. These findings highlight the potential of modulating higher-order RNA structures—such as the hairpin-G4 switch—to interfere with coronavirus replication [239].

## 9. Alternative RNA-Targeting Antiviral Approaches

Beyond ASOs and small molecules, additional modalities have broadened the landscape of RNA-targeted antivirals. For instance, the Adeno-Associated virus (AAV) delivered CRISPR–Cas13 systems using guide RNAs directed against conserved regions of the viral genome and have shown promise, efficiently cleaving and degrading EV71 RNA [240]. Aptamer-based strategies have also been explored in both HCV and SARS-CoV-2. In HCV, aptamers against subdomain IIId reduced IRES activity in vitro [191,192].

Using an in vitro systematic evolution of ligands by the EXponential enrichment (SELEX) approach starting from a diverse RNA library, Dantsu et al. enriched short single-stranded RNA molecules capable of folding into defined three-dimensional structures and binding their target with high affinity [241]. In this study, the selected aptamers were directed against the attenuator hairpin (AH) within the coronavirus-programmed −1 ribosomal frameshifting element, required for the balanced expression of the overlapping ORF1a/ORF1b region in all coronaviruses. The work identified and characterized a mirror image RNA aptamer that specifically recognizes the AH structure, illustrating the feasibility of designing aptamers that selectively engage the highly conserved viral RNA motifs involved in the critical steps of genome translation [241].

An innovative strategy to interfere with SARS-CoV-2 replication has focused on targeting the attenuator hairpin of the viral −1 ribosomal frameshifting element. A quinazoline-derived small molecule, C5, was identified that binds with high affinity to the refined attenuator hairpin, stabilizing its folded conformation and reducing frameshifting efficiency in cells [242]. Building on this interaction, the compound was further converted into a ribonuclease-targeting chimera (RIBOTAC) designed to recruit the cellular endoribonuclease RNase L, a key effector in the antiviral innate immune response, to the viral RNA (Figure 4F). This engineered ligand, C5-Chem-CLIP, not only recognizes the structural motif but also induces targeted degradation of the SARS-CoV-2 genome [242]. Another RIBOTAC-based strategy targeted the SL5 four-way junction in the 5′ UTR of the SARS-CoV-2 genome using coumarin-derived small molecules [243]. To define the ligand–RNA interaction site with high precision, Tang et al. developed a chemical-guided SHAPE-seq (cgSHAPE-seq) approach in which a modified acylating probe selectively reacted with the 2′-OH group at the ligand-binding site, leaving mutational signatures during reverse transcription that pinpointed the protected nucleotides. These coumarin derivatives were then repurposed to create RNA-degrading chimeras by replacing the acylating group with an RNase L-recruiting module. The resulting RIBOTAC (i.e., C64) efficiently degraded SARS-CoV-2 RNA in cell-based assays at low micromolar concentrations and inhibited replication of a live virus in lung epithelial cells without detectable toxicity. This work highlights how RNA structure-guided ligand mapping can directly inform the rational design of RNA-cleaving chimeras with antiviral activity [243].

Together, these studies highlight the wide range of possibilities for antiviral intervention when structured viral RNAs are used as therapeutic entry points. Targeting viral RNA elements offers inherent advantages—including a reduced likelihood of resistance and potentially lower toxicity—but substantial work is still needed to optimize these strategies. For ASOs, advances in chemical modification continue to improve stability and affinity. Modifications such as 2′-fluoro and 2′-O-methyl RNA, phosphorothioate linkages, phosphorodiamidate morpholinos (PMOs), and LNAs—including β-D- and α-L-LNA variants that constrain ribose geometry—have strengthened ASO performance, yet efficient delivery remains a major bottleneck [181,184]. For small molecules, the primary challenge lies in accurately defining the RNA secondary and tertiary structures they must engage. High-resolution structural information, combined with functional validation, is essential for identifying the most promising and conserved RNA motifs for therapeutic disruption. Functional RNA domains regulate key steps of the viral life cycle, including translation, replication, and encapsidation, and therefore represent compelling antiviral targets [36].

Efforts to systematically identify such structures have led to the development of computational and experimental frameworks. ScanFold, for example, detects regions of unusual thermodynamic stability indicative of conserved or functional RNA folds and has been applied to the ZIKV and HIV-1 genomes to reveal essential RNA structures [244]. Integrating approaches like ScanFold with SHAPE-based structural probing provides a powerful strategy for selecting optimal targets for therapeutic intervention. Equally important are the emerging techniques designed to pinpoint small-molecule binding sites directly on RNA, such as the recently developed structure-guided mapping method [243] and proximity-induced acylation approaches. Notably, reactivity-based transcriptome profiling has shown that many FDA-approved protein-targeted drugs unexpectedly bind numerous human RNAs in vivo—highlighting the need for high-throughput assessments of target specificity to avoid off-target effects and clinical failures [245].

Expanding these strategies to more complex RNA architectures, including pseudoknots and G-4, will be essential to develop better antiviral approaches. Recent high-throughput competitive binding assays have demonstrated their utility in identifying ligands for RNA tertiary motifs, such as the preQ1 riboswitch and SARS-CoV-2 G-4 [246]. Finally, not only local RNA structures but also long-range RNA–RNA interactions are increasingly recognized as crucial determinants of viral translation and genome replication, and these higher-order interactions may likewise represent valuable therapeutic targets [1].

## 10. Conclusions and Future Perspectives

Advances in chemical probing approaches, particularly SHAPE and its high-throughput derivatives, have profoundly expanded our understanding of viral RNA genomes. These methods have revealed that viral RNAs contain extensive and highly organized structural architectures that regulate multiple stages of the viral life cycle, including translation, replication, genome packaging, and interactions with host factors. SHAPE investigations of viral RNA genomes have confirmed previously characterized RNA structures while also uncovering numerous novel structural elements. Continued improvements in SHAPE methodologies and algorithms to predict SHAPE [247] will likely expand both the repertoire and information level of detectable RNA structures. For instance, SHAPE reagents react with the 2′-hydroxyl group of the ribose backbone of the four RNA nucleotides, compared to the lower resolution of DMS profiles. However, stacking interactions can make unpaired bases poorly reactive toward these reagents [30]. As a consequence, the interpretation of SHAPE reactivity can be challenging. Unpaired bases located in RNA hairpin loops can still be constrained by base-stacking interactions that make them weakly reactive to SHAPE probes [248]. Proper interpretation is therefore required to evaluate these cases. In addition, the formation of covalent adducts during chemical probing can potentially alter the underlying RNA structure being probed [249]. Recent developments in SHAPE chemistry have introduced probes that shorten the effective probing distance, enabling more precise RNA structure mapping [249].

Despite the rapid accumulation of genome-wide structural maps, a major challenge remains the functional interpretation of the numerous RNA elements identified through these approaches. In order to recover or infer models of RNA structures from these data, additional information often needs to be incorporated, either through computational methods or through multiple experimental approaches. Future studies will therefore need to integrate structural probing with systematic mutational analyses and viral fitness assays in order to distinguish biologically relevant regulatory motifs. The development of higher-throughput strategies to assess RNA structure functionality—particularly approaches that couple targeted mutagenesis with measurements of viral fitness—will be essential. In parallel, improved computational strategies will be necessary to prioritize candidate functional elements within the extensive structural landscapes of viral genomes. Because functional RNA structures tend to be evolutionarily conserved, phylogenetic analyses that reveal conserved base-pairing interactions can provide strong evidence for their functional relevance [250]. Moreover, while potential functional structures have been identified by different computational signatures, including low predicted entropy and high structural stability, additional layers of information could improve the predictive power of functional regions [34,46]. Following this, it is also foreseeable that the combination of experimental approaches with emerging RNA structure prediction strategies, including artificial intelligence-based methods, is expected to further expand our understanding of viral RNA structures [251].

In addition, chemical probing techniques inherently measure the RNA structure at the ensemble level. Because they interrogate populations of RNA molecules that may adopt multiple conformations, the resulting signals represent an average of the structural states present in the sample. RNA molecules often populate ensembles of alternative conformations rather than a single static structure. Recent experimental strategies, such as co-transcriptional SHAPE-seq [252] and SPET-seq [253], are beginning to reveal transient folding intermediates and low-abundance structural states, particularly when combined with computational methods to deconvolute the ensemble signal and infer alternative RNA conformations present within the experimental mixture [250]. Indeed, many viral RNA elements function as structural switches, and appropriate strategies are therefore required to characterize these dynamic conformational states. The HIV-1 RRE illustrates the functional relevance of RNA structural ensembles, as it can adopt two alternative conformations, with the five-way junction enabling binding of the Rev protein and promoting nuclear export of viral RNA [254].

Another important frontier lies in capturing RNA structural dynamics in physiological contexts, as RNA conformations are strongly influenced by the host cellular environment and by interactions with proteins, ribosomes, other RNAs and small molecules [82]. Expanding structural studies across multiple biological states—including virions, infected cells, and viral replication intermediates—will be essential to fully understand how RNA structure coordinates viral replication cycles. In particular, the integration of SHAPE-based techniques with strategies for mapping higher-order RNA interactions will provide important insights into the role of RNA structure throughout the viral life cycle. Additionally, integrating viral RNA structural probing with methods that map RNA–protein interactions will be important for understanding how RNA structure functions within ribonucleoprotein complexes. For example, combining SHAPE-based probing with crosslinking approaches such as CLIP can help identify RNA regions protected or altered by protein binding. Approaches such as footprinting and SHAPE-eCLIP, which compare RNA structure in the presence and absence of bound proteins, provide a strategy to map RNA–protein interaction interfaces at nucleotide resolution [255].

Viral RNA structures are increasingly recognized as important determinants of innate immune recognition and immune evasion. Viral RNAs are sensed by several pattern-recognition receptors, including toll-like receptors, RIG-I-like receptors, PKR, MDA5, and OAS proteins, which recognize distinct molecular features and operate through different antiviral pathways [256]. In the case of RIG-I, RNA recognition is promoted by features such as uncapped 5′-triphosphate or diphosphate ends, lack of 2′-O-methylation, and the presence of short duplex structures [257]. A well-characterized example is the Influenza A virus, in which the partially complementary 5′ and 3′ genomic termini form panhandle structures that contribute to RIG-I activation [258]. Additional viral RNA elements can modulate innate immune sensing, including U-rich regions in Hepatitis C virus RNA that stimulate RIG-I signaling [259], and the production of subgenomic flaviviral RNAs that sequester TRIM25 and impair RIG-I activation [18]. Beyond RIG-I, MDA5 is preferentially activated by long regions of double-stranded RNA, further highlighting the importance of RNA architecture in defining receptor specificity [260]. Together, these examples highlight that viral RNA architecture is not only a determinant of genome regulation and replication but also a key layer through which viral RNAs are discriminated from host transcripts and interpreted by innate immune sensors. A deeper understanding of how specific RNA structures engage host RNA-binding proteins and pattern-recognition receptors will be essential for elucidating host–virus interactions and may reveal new RNA-centered strategies for antiviral therapeutic development.

Importantly, the growing recognition of conserved functional RNA motifs across viral genomes also opens new opportunities for antiviral intervention. Structure-guided identification of accessible and functionally essential RNA elements may facilitate the development of RNA-targeted therapeutics, including antisense oligonucleotides and small molecules. Recent advances in RNA-targeted drug discovery highlight the growing potential of RNA as a therapeutic target and the expanding toolkit for identifying ligands that bind structured RNA elements [177]. In parallel, emerging deep-learning approaches that predict small molecule–RNA interactions without requiring prior knowledge of RNA tertiary structures may further accelerate the discovery of RNA-targeting compounds and could be particularly valuable for identifying ligands that bind structured viral RNAs [261]. Together, the continued integration of structural mapping, functional validation, computational analysis, and therapeutic design will be crucial for fully exploiting viral RNA architecture as a promising frontier for antiviral discovery. Ultimately, decoding the structural complexity of viral RNA genomes may redefine both our understanding of RNA virus biology and the strategies used to combat viral infections.

## Figures and Tables

**Table 1:** A summary of RNA structure probing studies using SHAPE methodologies. The table reports each analyzed virus that the SHAPE variant utilized, the chemical reagent that has been employed, and the main findings that the study obtained, together with its reference.

Virus	Technique	Reagent	Main Findings	References
**HCV**	SHAPE	NMIA	Map of long-range interactions of cis-acting replication elements	Tuplin et al. [39]
**PV**	SHAPE	NMIA	Identification of conserved RNA structures throughout the ORFs, within the 5′ UTR and IRES	Burrill et al. [40]
**HIV-2**	SHAPE	1M7	Structural characterization of the Rev response element (RRE) and RNA folding intermediates	Lusvarghi et al. [41]
**HIV-1**	hSHAPE	NMIA	High-resolution mapping of the first ~900 nt, showing how the 5′ UTR is highly structured and conserved	Wilkinson et al. [42]
**HIV-1**	hSHAPE	1M7	Proposed roles for RNA architecture in ribosome pausing and organizing unstructured regions	Watts et al. [29]
**HIV-1**	SHAPE-MaP	1M7, 1M6, NMIA	Identification of previously uncharacterized pseudoknots	Siegfried et al. [43]
**CHIKV**	SHAPE-MaP	1M7	Identification of 23 structured regions	Madden et al. [44]
**DENV**	SHAPE-MaP	1M7	Identification of 22 structural motifs involved in regulating genome architecture and viral replication	Boerneke et al. [7]
**WNV**	SHAPE-MaP in vivo and in vitro	NAI	Differences between in vitro and in vivo folding	Huston et al. [45]
**DENV, ZIKV**	SHAPE-MaP in vivo and in vitro	NAI	Differences between in vitro and in vivo folding with a focus on long-range interactions	Huber et al. [46]
**SARS-CoV-2**	icSHAPE	NAI-N_3_	Structural characterization of the viral genome in infected cells, including the identification of conserved structural regions across coronaviruses	Sun et al. [47]
**HCV**	SHAPE	1M7	Structural characterization of the IRES pseudoknot domain	Berry et al. [48]
**HCV**	SHAPE-Map	1M7	Identification of conserved structural elements implied in immune evasion	Mauger et al. [49]
**FMDV**	SHAPE (in vivo)	NAI	Identification of long-range interaction between IRES and 3′ UTR to modulate translation efficiency	Diaz-Toledano et al. [50]
**HCV**	SHAPE	Benzoyl cyanide (BzCN)	Functional characterization of 3′ UTR in IRES-dependent translation	Bai et al. [51]
**YFV**	SHAPE	NMIA	Discovered the existence of a pseudoknot required to stall XRN1 and enable the production of sfRNA	Silva et al. [52]
**SARS–CoV-2**	SHAPE-MaP	NAI	Functional characterization of s2m and its role in viral replication	Jiang et al. [53]
**HCV**	SHAPE	NAI	Described the regulatory motifs present in the coding region	Pirakitikulr et al. [54]
**DENV**	SHAPE	NMIA	Discovered elements implied in the cyclization of the genome	De Borba et al. [55]
**DENV**	SHAPE	NMIA	Characterization of the downstream of 5′ cyclization sequence (5′CS) pseudoknot	Liu et al. [56]
**DENV, ZIKV, JEV**	SHAPE	NMIA	Identification of 5′-UAR-flanking stem (UFS) element enabling genome cyclization and RdRp recruitment	Liu et al. [57]
**ZIKV**	icSHAPE	NAI-N3	Identification of functional structural elements and long-range intramolecular interaction specific to the Asian epidemic strains	Li et al. [58]
**IAV**	SHAPE	1M7	Identification of PSL2 involved in packaging	Hagey et al. [59]

**Table 2:** An overview of SHAPE reagents. The table summarizes commonly used SHAPE reagents, including their approximate half-lives, typical application conditions, and key experimental features.

Reagent	Half-Life	In Vivo/In Vitro	Characteristics
**NMIA**	260–430 s	Mainly in vitro	Classical SHAPE reagent; reacts with the 2′-OH group to form a 2′-O-adduct [37].
**1M7**	17 s	Both	Low nucleotide bias; particularly suitable for in vivo studies [93].
**1M6**	31 s	Mainly in vitro	Faster reactivity than NMIA [92].
**NAI**	~30 min	Mainly in vivo	Rapid RNA modification rate; shows bias against cytidine and guanosine. A quenching step is required [93].
**NAI-N_3_**	~30 min	Mainly in vivo	Rapid RNA modification rate; shows bias against cytidine and guanosine. A quenching step is required [93].
**B_Z_CN**	0.25 s	Mainly in vitro	Very fast reaction rate; inactivated by hydrolysis [99].

## Data Availability

No new data were created or analyzed in this study. The SHAPE data used for the profiles in Figure 2A were downloaded from the original manuscripts.
